# A case–control study of factors associated with SARS-CoV-2 infection among healthcare workers in Colombia

**DOI:** 10.1186/s12879-021-06581-y

**Published:** 2021-08-27

**Authors:** Merida Rodriguez-Lopez, Beatriz Parra, Enrique Vergara, Laura Rey, Mercedes Salcedo, Gabriela Arturo, Liliana Alarcon, Jorge Holguin, Lyda Osorio

**Affiliations:** 1 Pontificia Universidad Javeriana Seccional Cali, Calle 18 No.118 - 250 Edificio Raúl Posada S.J. Tercer Piso, Cali, Colombia; 2 Universidad del Valle, Cali, Colombia; 3 Secretaria de Salud Pública Municipal de Cali, Cali, Colombia

**Keywords:** COVID-19, SARS-CoV-2, Risk factors, Healthcare workers

## Abstract

**Background:**

Healthcare Workers (HCW) are repeatedly exposed to SARS-CoV-2 infection. The aim of this study was to identify factors associated with SARS-CoV-2 infection among HCW in one of the largest cities in Colombia.

**Methods:**

We conducted a case–control study, where cases had a positive reverse transcription-polymerase chain reaction and controls had a negative result. Participants were randomly selected and interviewed by phone. Analyses were performed using logistic regression models.

**Results:**

A total of 110 cases and 113 controls were included. Men (AdjOR 4.13 95% CI 1.70–10.05), Nurses (AdjOR 11.24 95% CI 1.05–119.63), not using a high-performance filtering mask (AdjOR 2.27 95% CI 1.02–5.05) and inadequate use of personal protective equipment (AdjOR 4.82 95% CI 1.18–19.65) were identified as risk factors. Conversely, graduate (AdjOR 0.06 95% CI 0.01–0.53) and postgraduate (AdjOR 0.05 95% CI 0.005–0.7) education, feeling scared or nervous (AdjOR 0.45 95% CI 0.22–0.91), not always wearing any gloves, caps and goggles/face shields (AdjOR 0.10 95% CI 0.02–0.41), and the use of high-performance filtering or a combination of fabric plus surgical mask (AdjOR 0.27 95% CI 0.09–0.80) outside the workplace were protective factors.

**Conclusion:**

This study highlights the protection provided by high-performance filtering masks or double masking among HCW. Modifiable and non-modifiable factors and the difficulty of wearing other protective equipment needs to be considered in designing, implementing and monitoring COVID-19 biosafety protocols for HCW.

## Introduction

Over 55 millions of people were infected worldwide by the Severe Acute Respiratory Syndrome Coronavirus 2 (SARS-CoV-2) in 2020 [1]. In previous coronavirus pandemic outbreaks, SARS-CoV-1 in 2003 and the Middle East Respiratory Syndrome in 2012, between 10 and 20% of infected people were Healthcare Workers (HCW) [2, 3]. In the current pandemic, the prevalence among HCW varies between countries from 2 to 30% [4]. The Coronavirus Disease 2019 (COVID-19) caused by SARS-CoV-2, affects people’s lives and threatens their biological [5, 6], physiological [7, 8], family and social health [9, 10]. HCW are repeatedly exposed to the virus leading to an increased risk of the disease [11] and sequelae [12] compared to the general population. Hence, COVID-19 could reduce the workforce availability to respond to this emergency.

The first case of SARS-CoV-2 in Colombia was reported in March 2020. Seven months later, the Colombian National Institute of Health has informed over 16,500 infected HCW, most of whom were associated to the workplace [13]. In a descriptive study of HCW in Cali, one of the largest cities in Colombia, 65% of infections were related to the workplace and the most affected were women and nursing assistants [14]. To date, there is scarce evidence in Latin America, concerning risk factors for the infection particularly among HCW, who are exposed to both, workplace and community transmission. Studies are mostly from Asia, Europe and North America [15–18]. They have focused on nurses and medical staff, and they have mainly evaluated the presence of symptoms and the exposures to occupational factors, including aerosol-generating procedures [15]. Cultural differences and availability of resources between countries and institutions, limit a direct extrapolation of previous findings. Less is known about the effect of factors related to potential community transmission or the risk among other hospital workers. Moreover, there is controversy abound the appropriate types of masks for HCW in community settings [19]. Therefore, the aim of this study was to determine the factors associated with SARS-CoV-2 infection among HCW in Cali, Colombia.

## Subjects and methods

### Study design

We conducted a case–control study in HCW who served in health care institutions in Cali, Colombia. Participants were identified by merging the database of positive reverse transcription-polymerase chain reaction (RT-PCR) results with the routine surveillance system of COVID-19 (event code 346) or acute respiratory infections (event codes 345 and 348), who were reported with or without symptoms (as part of cluster investigations), between June 10^th^ and July 25^th^, 2020. This time framework matches the first peak of the epidemic curve in Cali [20]. Cases and controls were randomly (simple random sampling without replacement) selected from those identified as HCW with a positive and negative test, respectively. The outcome status was confirmed with the database of epidemiological investigation of COVID-19 in health care facilities compiled by the local health authorities of Cali and during the telephone interview. This strategy ensures a representative sample of different health care institutions independently of size, patient type, care level, management or service provided. Sample size was estimated as 111 participants for each group with 80% power, 95% level of confidence, 18% of exposure among controls, Odds Ratio (OR) of 2.5, 1:1 allocation ratio, and 10% of withdrawal.

HCW were defined as those working in healthcare environments regardless of whether they were directly or indirectly involved in clinical activities such driving an ambulance or worked in a hospital or in homecare. Potential participants were contacted by phone and eligibility criteria were confirmed (18 years or older, not being pregnant or having a coagulopathy, and working in a health care institution that have the potential to assist COVID-19 patients, or being in contact before they had a RT-PCR test with infectious materials such as body fluids and contaminated surfaces and supplies). The study protocol was part of the public health research to face the pandemic and was revised by the Universidad Javeriana Cali Ethics Committee. Inform consent was obtained online for all participants.

### Data collection

Data was collected by two trained researchers via telephone and using a structured questionnaire. The questionnaire included modifiable and non-modifiable factors: sociodemographic, clinical and lifestyle factors referred to six months before the test result, psychological factors referred to one month before the test. Occupational, exposure to COVID-19 cases, social behavior and personal protection equipment (PPE) factors referred to two weeks before the RT-PCR test. Feeling scared or nervous or having insomnia were evaluated by a five-point Likert scale, and further dichotomized as never or anytime. Height, weight, and compliance to recommended PPE use were self-reported. The exposure to a positive person was evaluated by the question: “To your knowledge, were you in contact with a person diagnosed with COVID-19, at least 2 weeks prior to the test?” A high-performance filtering mask was considered as the use of N95, P100 or M3. The frequency of use of each PPE at work were classified as always wearing them or not. Self-perception of the adequate use of PPE was evaluated as many times, sometimes or few times. The use of medicines for prophylaxis purposes included hydroxychloroquine and ivermectin. Vitamins, nutritional supplements, and hormonal contraceptives, usually taken for a long period were also included. Interviewers were blinded to the case status. At the end of each interview, blindness was broken to confirm the status of each participant as to prevent potential misclassification bias due to controls having a positive test after their report to the surveillance system.

### Statistical analysis

Normality assumption was checked using Shapiro Wilk test. Then, study groups were described and compared using median (interquartile range) and relative frequencies for quantitative and qualitative variables, respectively. Body Mass Index (BMI) was estimated from self-reported weight and height and categorized as obese (≥ 30 kg/m^2^), overweight (25 to < 30 kg/m^2^) and not overweight nor obese (< 25 kg/m^2^). Epidemiological weeks were calculated based on the date of the test result. To account for correlation among exposures in multiple analysis, new variables were defined. For example, the use of surgical caps, goggles/face shields, and gloves were grouped as single PPE. As HCW may have work in more than one hospital area, these were classified according to risk as “high-risk” if working in COVID-19-designated zones and any of emergency room, inpatient ward or intensive care unit (ICU), as “middle risk” if did not work in a COVID-19-designated zone but in emergency or ICU, and as “low risk” if did not work in COVID-19-designated zone nor emergency nor ICU.

Mann–Whitney *U*-test and Chi-Square or Fisher test were used for comparisons as appropriate. Multiple Logistic regression models were fitted using the backward strategy and the likelihood ratio test. A variable remained in the model when partial F had a *P* ≤ 0.10, when confounding effect was observed, or by its clinical relevance on the outcome (i.e.; epidemiological weeks and hospital area). Model fit was evaluated by Hosmer and Lemeshow test. Calibration, specificity, and collinearity was also checked. The final model was selected considering the highest explicative ability measured by PseudoR^2^. Analyses were performed using Stata version 15 (StataCorp. LP, College Station,TX).

## Results

**Fig. 1:**
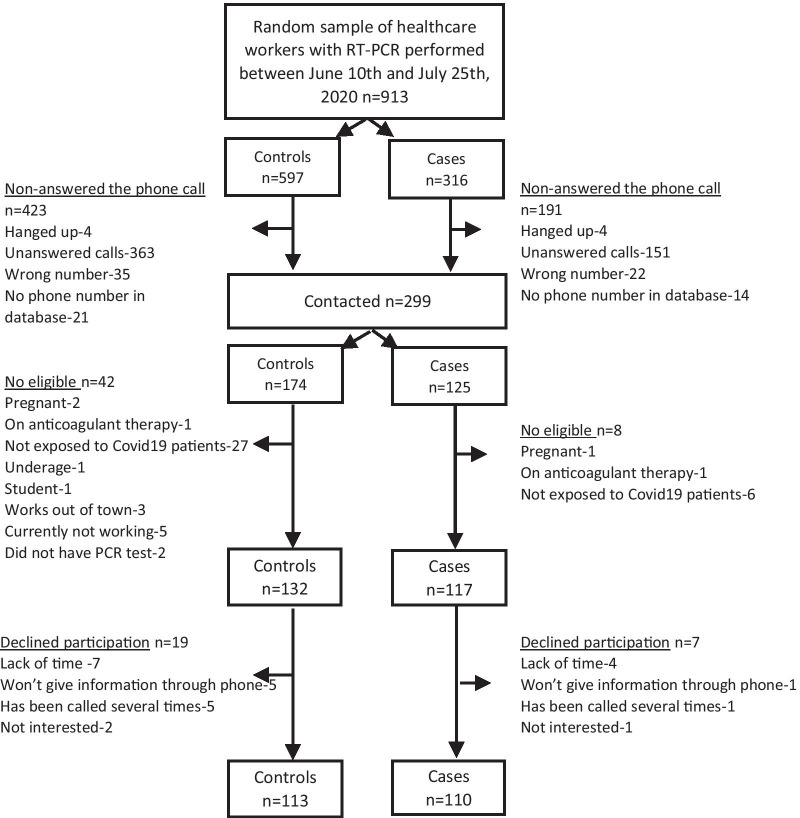
Flowchart of the study participants

The flow diagram of the study population is shown in Fig. 1. Among those contacted that met the eligibility criteria, 5% of cases and 14% of controls declined to participate, resulting in a final sample of 110 cases and 113 controls. RT-PCR was ordered because of symptoms in 59.2% of participants and the remaining 40.8% as part of contact tracing or institutional screening. At the time of the interview, all HCW reported to wear some type of facemask in both the institutional and community settings. Oral contraceptives were the most common type of hormonal contraception (82%). Differences between cases and control are shown in Tables 1, 2 and 3. Among females, the difference between cases and controls in the use of hormonal contraceptives was observed mainly in symptomatic women (OR = 2.05 95% CI 0.75–5.64).

**Table 1:** Association between sociodemographic, clinical, lifestyle and psychological characteristics with SARS-CoV-2 infection among HCW

Characteristics	Negative RT-PCR n = 113	Positive RT-PCR n = 110	Unadjusted OR (95% CI)	P value
Sociodemographic				
Age, years	30 (26–34)	30 (27–34)	0.98 (0.95–1.02)	0.40
Gender, n (%)				
Female, not taking hormonal contraceptives	82 (72.57)	57 (51.82)	Ref.	
Female, taking hormonal contraceptives	11 (9.73)	16 (14.55)	2.09 (0.90–4.84)	0.08
Male	20 (17.70)	37 (33.64)	2.66 (1.40–5.04)	< 0.01
Socioeconomic stratum, n (%)				
Low (1–2)	37 (32.74)	31 (28.18)		
Middle (3–4)	56 (49.56)	68 (61.82)	1.44 (0.80–2.63)	0.22
High (≥ 5)	20 (17.70)	11 (10)	0.65 (0.27–1.57)	0.35
Race, n (%)				
White	37 (32.74)	31 (28.18)	Ref.	
Mestizo	63 (55.75)	62 (56.37)	1.17 (0.65–2.12)	0.59
Afrocolombian	13 (11.50)	17 (15.45)	1.56 (0.66–3.71)	0.31
Highest educational level, n (%)				
Non-college education	50 (44.25)	62 (56.36)	Ref.	
College graduate	40 (35.39)	36 (32.73)	0.72 (0.40–1.30)	0.28
Postgraduate education	23 (20.35)	12 (10.91)	0.42 (0.19–0.93)	0.03
Marital status, n (%)				
Married or in common law	41 (36.28)	45 (40.91)	Ref.	
Single or Divorced	72 (63.72)	65 (59.09)	0.82 (0.48–1.41)	0.48
Number of members in household, n (%)				
≤ 1	24 (21.24)	37(33.64)	Ref.	
> 2	89 (78.76)	73 (66.36)	0.53 (0.29–0.97)	0.04
Epidemiological weeks, weeks	27 (25–28)	27 (26–28)	1.11 (0.95–1.30)	0.18
Clinical, Life Style and Psychological risk factors				
Obesity, n (%)				
Not overweight nor obese	53 (47.75)	44 (40.37)	Ref.	
Overweight	41 (36.94)	49 (44.95)	1.44 (0.81–2.56)	0.22
Obese	17 (15.32)	16 (14.68)	1.13 (0.51–2.50)	0.76
Chronic disease, n (%)				
No	100 (88.50)	95 (86.36)	Ref.	
Yes (HBP, DM, Asthma Hypothyroidism)	13 (11.50)	15 (13.64)	1.21 (0.54–2.68)	0.63
Medication intake, n (%)				
Vitamins or supplements (yes)	20 (26.55)	20 (18.88)	0.61 (0.32–1.16)	0.14
Chloroquine or Ivermectin (yes)	14 (12.39)	7 (6.36)	0.48 (0.19–1.24)	0.13
Smoking habit, n (%)				
No	92 (81.42)	85 (77.27)	Ref.	
Former or current smoker	21 (18.58)	25 (22.73)	1.28 (0.67–2.47)	0.44
Alcohol intake, n (%)				
Never	32 (28.32)	44 (40)	Ref.	
Monthly intake	71 (62.83)	57 (51.82)	0.58 (0.33–1.04)	0.07
Weekly intake	10 (8.85)	9 (8.18)	0.65 (0.24–1.79)	0.41
Feeling scared or nervous, n (%)				
Never	41 (36.28)	57 (51.82)	Ref.	
Anytime	72 (63.72)	53 (48.18)	0.53 (0.31–0.90)	0.02
Insomnia, n (%)				
Never	49 (43.36)	52 (47.27)	Ref.	
Anytime	64 (56.64)	58 (52.73)	0.85 (0.50–1.45)	0.56

*HBP* High Blood Pressure, *DM* Diabetes Mellitus

**Table 2:** Association between occupational, exposure and social behavior factors and SARS-CoV-2 infection among HCW

Characteristics	Negative RT-PCR n = 113	Positive RT-PCR n = 110	Unadjusted OR (95% CI)	*P* value
Occupation, n (%)				
Nursing Assistant	31 (27.43)	38 (34.55)	Ref.	
Nurse	18 (15.93)	19 (17.27)	0.86 (0.39–1.91)	0.71
Physician	25 (22.12)	18 (16.36)	0.58 (0.27–1.27)	0.17
Other healthcare professionals^a^	12 (10.62)	10 (9.09)	0.68 (0.26–1.78)	0.43
Technicians	12 (10.62)	15 (13.64)	1.01 (0.42–2.49)	0.96
Administrative Staff	15 (13.27)	10 (9.09)	0.54 (0.21–1.37)	0.19
Workplace risk area, n (%)				
Worked in a COVID-19 zone (yes)	47 (41.59)	52 (47.27)	1.25 (0.74–2.13)	0.39
Worked in a high-risk area (yes)	54 (47.79)	70 (63.64)	1.91 (1.12–3.27)	0.02
Night shifts, n (%)				
No	61 (53.98)	42 (38.18)	Ref.	
Yes	52 (46.02)	68 (61.82)	1.89 (1.11–3.23)	0.02
Face-to-face contact with a positive case, n (%)				
No contact	29 (25.66)	29 (26.36)	Ref.	
Yes, family or work colleagues	42 (37.16)	22 (20)	0.52 (0.25–1.09)	0.08
Yes, patients	42 (37.17)	59 (53.64)	1.40 (0.73–2.69)	0.30
Shared spaces at work, n (%)				
Cafeteria (yes)	42 (37.17)	38 (34.55)	0.89 (0.52–1.54)	0.68
Resting places (yes)	14 (12.39)	22 (20)	1.77 (0.85–3.66)	0.13
Means of transportation to work, n (%)				
None or private	84 (74.34)	77 (70)	Ref.	
Taxi, Ridesharing, Public transportation	29 (25.66)	33 (30)	1.24 (0.69–2.23)	0.47
Outings besides work, n (%)				
To the bank (yes)	14 (12.39)	23 (20.91)	1.87 (0.91–3.86)	0.09
To the supermarket/store (yes)	81 (71.68)	66 (69.09)	0.59 (0.34–1.03)	0.07

^a^mainly dentists and physiotherapists

**Table 3:** Association between personal protection equipment at work and in the community and SARS-CoV-2 infection among HCW

Characteristics	Negative RT-PCR n = 113	Positive RT-PCR n = 110	Unadjusted OR (95% CI)	*P* value
Use of High-performance filtering mask, n (%)				
Always	51 (45.13)	47 (42.73)	Ref.	
Not always or another mask	62 (54.87)	63 (57.27)	1.10 (0.65–1.86)	0.72
Use of Gloves, n (%)				
Always	58 (51.33)	72 (65.45)		
Not always	55 (48.67)	38 (34.55)	0.56 (0.32–0.95)	0.03
Use of Face shield or goggles, n (%)				
Always	65 (57.52)	77 (70)		
Not always	48 (42.48)	33 (30)	0.58 (0.33–1.01)	0.05
Use of Surgical cap, n (%)				
Always	52 (46.02)	73 (66.36)	Ref.	
Not always	61 (53.98)	35 (33.64)	0.43 (0.25–0.74)	< 0.01
Used PPE properly, n (%)				
Many times	108 (95.58)	97 (88.18)	Ref.	
Sometimes/few times	5 (4.42)	13 (11.82)	2.89 (1–8.42)	0.05
Training PPE support, n (%)				
None	20 (17.70)	9 (8.18)	Ref.	
< 2 h	60 (53.10)	62 (56.36)	2.30 (0.97–5.44)	0.06
≥ 2 h	33 (29.20)	39 (35.45)	2.63 (1.05–6.54)	0.04
Type of facemask outside of work, n (%)				
Surgical mask	69 (61.06)	79 (71.82)	Ref.	
High-performance or fabric plus surgical mask	27 (23.89)	12 (10.91)	0.39 (0.18–0.82)	0.01
Only fabric mask	17 (15.04)	19 (17.27)	0.98 (0.47–2.02)	0.95

Modifiable and non-modifiable risk factors remained in the multivariate model as shown in Table 4. The use of a high-performance mask or a combination of fabric and surgical mask outside the workplace showed a protective effect (AdjOR = 0.27 95% CI 0.09–0.80). Not wearing any of surgical caps, face shields/goggles or gloves (AdjOR = 0.10 95% CI 0.02–0.41) and feeling scared or nervous (AdjOR = 0.45 95% CI 0.22–0.91) were also protective. On the contrary, not always wearing high-performance mask within the workplace (AdjOR = 2.27 95% CI 1.02–5.05) and not using PPE properly (AdjOR = 4.82 95% CI 1.18–19.65) were positive associated with the infection. Male gender (AdjOR = 4.13 95% CI 1.70–10.05) and being nurse AdjOR = 11.24 95% CI 1.05–119.63) increased the risk, while college graduate AdjOR = 0.06 95% CI 0.01–0.53) and postgraduate education (AdjOR = 0.05 95% CI 0.005–0.47) reduced the risk of a positive RT-PCR.

**Table 4:** Multiple regression model of factors associated with SARS-CoV-2 infection in HCW. (Model also adjusted by epidemiological week and hospital area)

Characteristics	Adjusted OR (95% CI)	P value
Gender		
Female, not taking hormonal contraceptives	Ref.	
Female, taking hormonal contraceptives	2.72 (0.91–8.13)	0.07
Male	4.13 (1.70–10.05)	< 0.01
Highest educational level		
Non-college education	Ref.	
College graduate	0.06 (0.01–0.53)	0.01
Postgraduate education	0.05 (0.005–0.47)	< 0.01
Number of members in the household		
≤1	Ref.	
> 2	0.47 (0.21–1.04)	0.06
Feeling scared or nervous		
Never	Ref.	
Anytime	0.45 (0.22–0.91)	0.03
Alcohol intake		
Never	Ref.	
Monthly intake	0.50 (0.23–1.07)	0.08
Weekly intake	0.58 (0.16–2.16)	0.42
Occupation		
Nursing Assistant	Ref.	
Nurse	11.24 (1.05–119.63)	0.04
Physician	8.36 (0.81–85.36)	0.07
Other healthcare professionals^a^	11.44 (0.88–148.70)	0.06
Technicians	2.49 (0.71–8.67)	0.15
Administrative Staff	3.99 (0.91–17.66)	0.07
Use of High-performance filtering mask		
Always	Ref.	
Not always or another face mask	2.27 (1.02–5.05)	0.04
Always wear caps, face shield/goggles or gloves		
Yes, all three of them	Ref.	
Yes, only two	0.69 (0.25–1.96)	0.49
Yes, only one	0.35 (0.13–0.98)	0.05
None	0.10 (0.02–0.41)	< 0.01
Used PPE properly		
Yes	Ref.	
No	4.82 (1.18–19.65)	0.03
Type of facemask outside of work		
Surgical mask	Ref.	
High-performance or fabric plus surgical mask	0.27 (0.09–0.80)	0.02
Only fabric mask	0.84 (0.32–2.20)	0.73
Training PPE support		
None	Ref.	
< 2 h	2.73 (0.83–8.92)	0.10
≥ 2 h	3.42 (0.97–12.01)	0.06

^a^mainly dentists and physical therapists

## Discussion

This study identified modifiable and non-modifiable factors associated to a positive RT-PCR among HCW. Particularly, a greater protective effect of high-performance masks, or double masking outside the workplace was observed when compared to other types. Conversely, surgical caps, face shields/goggles and gloves were found to increase risk. Psychological factors that prevented being overconfident about SARS-CoV-2 transmission were protective. For non-modifiable factors, male gender increased the risk while higher level of education was protective.

Concerning face-masks, those HCW always-wearing high-performance filtering masks had a better protection when compared to those wearing them occasionally or wearing other types of facemasks. This protective effect is controversial in the literature, with results suggesting greater [21], similar [22] or even lower [23] protection compared to surgical masks. Different types of masks, manufacturer standards, and the evaluation of potential confounders may explain discordances between studies. In addition, there is not a clear recommendation for the type of mask that HCW need to wear outside the workplace [19, 24]. In line with previous studies [25, 26], our results suggest that fabric and surgical masks performed similarly, while wearing high-performance filtering masks or a combination of fabric plus surgical mask reduces the risk of infection compared to the use of surgical mask exclusively. Therefore, HCW could be advised to wear high-performance mask even when they are not directly taking care of COVID-19 patients, or in case of a shortage, low resource settings or high cost of high-performance masks, a combination of fabric plus surgical mask as an alternative.

Controversially, our study reported a greater risk among those who always wore face shields/goggles, gloves and surgical caps. In this regard, the evidence is limited [24] and the statistically significant protective effect disappears after covariates adjustment [27]. A false sense of safety resulting in self-contamination, sharing reusable PPE without appropriate disinfection protocols, or relaxing their use [28–30] could explain this result. In any case, emphasis needs to be given to the proper use of PPE during and after patient´s care, as previously stated [15, 31–33].

Another modifiable psychological factor showing a protective effect was feeling scared or nervous. Despite the fact that we did not evaluate the source of stress, anxious individuals are less confident in their abilities to managing threated situations [34]. Therefore, they are more sensitive to feedback and to be hypervigilant in monitoring their surroundings and themselves which leads to strategic actions to avoid harm [35]. Whether this apparent protective effect will persist through the duration of the pandemic needs to be elucidated.

Non-​modifiable risk factors included sex, education and occupation.Our results support a greater risk of having a positive RT-PCR among men. The testosterone suppression effect on the innate immune responses [36], the differential expression of ACE2 between males and females [37], and a better compliance among women with biosafety measures [38] could explain the gender differences in COVID-19 susceptibility. Notably, we observed a differential but no significant risk among women according to the use of hormonal contraceptives, which requires further evaluation. The greater risk among less-educated adults compared to university graduated is consistent with a previous report [39]. Our study reports a greater risk among nurses when compared to nursing assistants; however, the precision of this estimation was low. Despite these factors are not modifiable, some strategies focusing on high risk groups could be implemented to reduce their risk, e.g. special training and monitoring for men and less educated groups.

To prevent misclassification bias, interviewers were masked to the participant´s case or control status. Although we did not quantify the possible effect of recall bias, phone questionnaires have been used in other pandemics [40] and are as valid as face-to-face interviews for collecting behavioural information [41, 42]. Moreover, we expect recall bias to be non-differential given that the time between the RT-PCR results and the interview were similar between groups. Self-report of anthropometric measures has been found to be accurate in terms of weight classification [43, 44]. The reasons for declining participation were similar between groups and were mainly related to availability (in terms of time), which made selection bias unlikely. Residual confounding could be present due to unmeasured variables such as quality of training, doffing practices, or the prevalence of the infection in the place of residence. In addition, residual confounding could be due to remaining differences in variables such as the type of hormonal contraceptives and the number of mask layers. Our results should not be extrapolated to the general population because health care workers are likely to behave differently regarding PPE use and risk of infection.

In conclusion, modifiable and non-modifiable factors were associated to SARS-CoV-2 infection among HCW, independent of the level of exposure. High-performance masks or double masking, adequate use of PPE and feeling scared or nervous were protective factors. In addition, gender, level of education along with occupational characteristics, were also associated with the risk of infection and need to be considered when planning public health and health care facilities prevention strategies.

## Data Availability

The datasets used and/or analysed during the current study are available from the corresponding author on reasonable request.

## Abbreviations

ACE: Angiotensin Converting Enzyme
Adj OR: Adjusted Odds Ratio
CI: Confidence Interval
COVID-19: Coronavirus Infection Disease 2019
DM: Diabetes Mellitus
HCW: Health Care Worker
HBP: High Blood Pressure
PPE: Personal Protection Equipment
ICU: Intensive Care Unit
OR: Odds Ratio
RT-PCR: Reverse Transcription-Polymerase Chain Reaction
SARS-CoV-2: Severe Acute Respiratory Syndrome Coronavirus 2
